# Straw Biochar and Graphite Oxide Enhanced External Pressure Ultrafiltration for Leaded Wastewater Treatment

**DOI:** 10.3390/toxics12070500

**Published:** 2024-07-10

**Authors:** Fan Yang, Liang Pei, Zhenggang Wang, Jia Duo

**Affiliations:** 1 Xinjiang Institute of Ecology and Geography, Chinese Academy of Sciences, Urumqi 830011, China; yangfan@ms.xjb.ac.cn (F.Y.); duojia2017@ms.xjb.ac.cn (J.D.); 2 Key Laboratory of Xinjiang Coal Resources Green Mining (Xinjiang Institute of Engineering), Ministry of Education, Urumqi 830023, China; 3 Institute of Geographical Sciences and Resources, Chinese Academy of Sciences, Beijing 100101, China; 4 College of Resources and Environment, University of Chinese Academy of Sciences, Beijing 100049, China; 5 Xinjiang Research Center for Natural Resources Development, Urumqi 830002, China

**Keywords:** straw biochar, graphene oxide, external pressure ultrafiltration, leaded wastewater

## Abstract

In order to solve the problem of the low treatment efficiency of wastewater containing heavy metals in mining areas, straw biochar and graphene oxide enhanced external pressure ultrafiltration (SGU) was used to treat wastewater containing high concentrations of Pb^2+^. The operation parameters such as pH and temperature were optimized, and the removal efficiency of COD_Cr_, NH_3_-N, turbidity and Pb^2+^ via SGU, straw biochar ultrafiltration (SU), ultrafiltration (UF), and conventional treatment (CT) were systematically investigated. The results showed that the pH and temperature of polluted water were 4.8–5.2 and 21–30 °C, respectively, the average removal rates of COD_Cr_, NH_3_-N, turbidity and Pb^2+^ by SGU reached 91%, 97%, 98% and 95%, respectively, and the removal effect was better than that of other processes. In addition, under the backwash conditions of clean water, weak acid, and weak alkali, the membrane flux recovered 65%, 88%, and 89% of the new membrane, respectively. This study provides scientific and theoretical support for the advanced treatment of polluted water in mining areas.

## 1. Introduction

The treatment of micro-polluted water originating from mining areas and the reuse of miscellaneous water are common concerns of the industry and mining industry. The water quality changes in micro-polluted water, especially water with many unique substances, create difficulties for traditional water treatment methods to cope with. Owing to their advantages of a high molecular weight of interception, low operating pressure, and low operating cost, ultrafiltration membranes have been widely used in the field of water treatment. However, practical wastewater in mining areas contains some organic matter or small dissolved molecules, which cannot be removed by an ultrafiltration membrane. At present, combining an ultrafiltration membrane with other methods is an efficient way to solve this problem [1,2,3]. The contaminants existing in polluted water do harm to human beings [4]. To overcome the threat, a series of conventional wastewater treatment technologies such as chemical precipitation, adsorption, electrodialysis, ion exchange, membrane filtration, flocculation, and electrochemical precipitation are used to remove heavy metals and organic pollutants [5].

A variety of negatively charged functional groups grow on the biochar surface. Through coordination, specific complexes can be formed between these functional groups and heavy metal ions in polluted water, which can anchor heavy metal ions on the surface of biochar, thus providing an effective new way for the treatment of heavy metal polluted water [6,7]. Due to its characteristics of loose, porous, and rich plant fibers, waste straw can be used as a raw material for biochar preparation. If waste straw residue is used as raw material for biochar preparation and supplemented by a composite modification of novel graphene adsorption materials, its adsorption performance can be maximized, which is of great significance for the resource utilization of waste straw residue and graphite waste [8]. Owing to their large specific surface area and strong adsorption capacity, biochar and GO (graphene oxide) are widely used in the preparation of chemical products and wastewater treatment. According to previous studies, pores exist in biochar particles, and the GO is a 2-D material. After the preparing process, GO is anchored on the surface of biochar, which promotes the formation of a three-dimensional interleaved network between GO and biochar [9,10,11,12]. The composite not only has strong adsorption but also can better remove pollutants with a particle size larger than the pore size [13,14,15,16,17,18,19,20]. Ma et al. [21] prepared nano-biochar to remove Cd^2+^ from water, the results showing that the maximum adsorption amount of Cd^2+^ was 52.00 mg/g under optimized conditions. Zhang et al. [22] studied the adsorption properties of Pb^2+^ and atrazine by a biochar-supported reduced graphene oxide composite and found that the adsorption capacities of the materials was 26.10 mg/g for Pb^2+^ and 67.55 mg/g for atrazine. Meanwhile, the adsorption performance of the composites was stable after four times of being used. Additionally, biochar/GO also can be used to adsorb heavy metal ion from soil. Carneiro et al. [23] prepared livestock feces biochar (PLB) and PLB-GO and studied their adsorption effects on copper (Cu) and zinc (Zn) in soil. The adsorption capacity of Cu and Zn by PLB-GO is 16.2% and 17.7% higher than that of original PLB, respectively. Moreover, the adsorption of copper and zinc via PLB and PLB-GO is relatively stable. To the best of our knowledge, reports that combine biocharGO and polyvinylidene fluoride (PVDF) to treat actual wastewater are seldom.

Therefore, in this study, biochar–GO was prepared using waste straw residue and natural graphite as raw materials and was combined with a PVDF external pressure ultrafiltration membrane module to treat the contaminated water in a mining area. As Pb^2+^ concentration in the selected study area is much greater than the standard, the removal of COD, NH_3_-N, turbidity, and Pb^2+^ was selected to evaluate the performance of the SGU system. The operation parameters of the straw biochar–graphene oxide–ultrafiltration (SGU) system were optimized, and the removal ability of SGU, straw biochar–ultrafiltration (SU), ultrafiltration (UF) and conventional treatment (CT) was compared. The study provides a reference for the advanced treatment of micro-polluted water with more unique substances in a mining area.

## 2. Materials and Methods

### 2.1. Materials and Reagents

GO was bought from Xiamen Haibiao Co., Ltd. (Xiamen, China) and Xianfen Nami Co., Ltd. (Nanjing, China). The raw water originated from a metal mining area in Liaoning Province, and its parameters are displayed in Table 1.

### 2.2. Preparation of SB/GO

Corn straw was smashed and filtered through a mesh. The obtained powder was washed using water several times and dried at 60 °C for 12 h. Then, we put the dried sample into a furnace, and the temperature was kept at 300 °C for 2 h. When the furnace cooled to room temperature, the solid products were collected. For SB/GO preparation, the operation process is the same as the SB preparation, but we introduce GO into the furnace before the heating process begins (V_SB_:V_GO_ = 20:1).

### 2.3. Equipments and Methods

**Figure 1:**
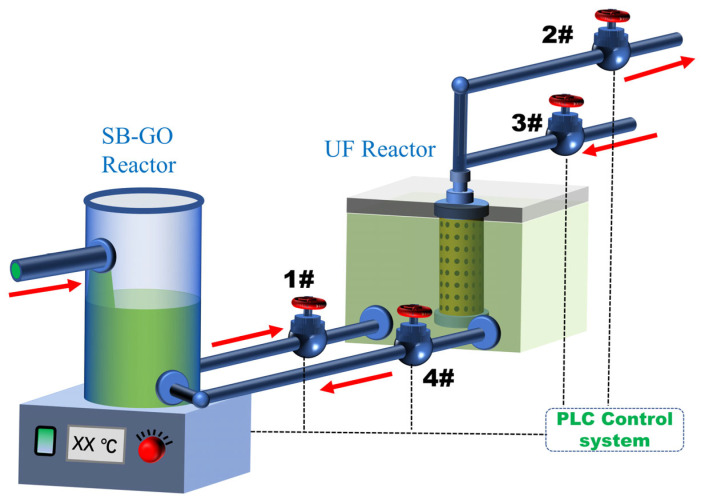
The diagram of equipment.

A diagram of the equipment is shown in Figure 1. The SB-GO reactor in the system is a cylindrical container (diameter 30 cm, height 80 cm), and the UF reactor in the system is a cuboid container (118 cm × 46 cm × 67 cm) with an effective volume of 310 L. A PVDF hollow-fiber ultrafiltration membrane (Mitsubishi Company, Tokyo, Japan) served as the membrane component. The intercepted molecular weight, membrane aperture, outer diameter, and effective surface area were 100,000, 0.12 µm, 0.2 mm, and 11.8 m^2^, respectively.

Mining wastewater comes from a metal mining area in Liaoning Province. Mining wastewater is filtered by a sieve mesh (1 mm) and entered the SB/GO reactor, stirring for 20–40 min. Mining wastewater was cycled in the system through pumps 1 and 4. Then, pump 1 was shut off and pump 2 turned on, and water entered the UF reactor to begin the UF process. The stirring rate, pH, and temperature of mining wastewater were optimized to achieve the best performance of the system. The mixing speed is 150–180 r/min. There was no artificial blowdown during the process. Meanwhile, to compare the treatment performance of the SGU process, SU, UF and CT, the same mining wastewater was treated by these 4 processes, respectively. Additionally, when the membrane flux was close to 0, it was washed using clear water, weak acid, or weak base backwash every 3 h via controlling pumps 2 and 3. The whole device is controlled by a PLC automatic control system. The operating pressure of the membrane module is 25 kPa under a normal state, and the maximum operating pressure is 68 kPa. The design maximum daily water yield of the system is 10 m^3^. The reactor ran well from March 2021 to August 2021.

## 3. Results and Discussion

### 3.1. FT-IR Spectra of Samples

**Figure 2:**
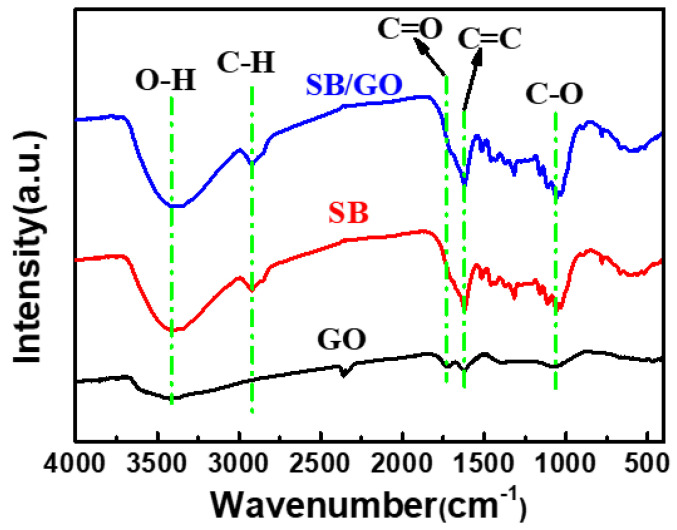
FT-IR spectra of GO, SB, and SB/GO.

The FT-IR spectra of GO, SB, and SB/GO are shown in Figure 2. As shown in the figure, the peak at ~3400 cm^−1^ is the stretching vibration of O-H, the peak at ~2900 cm^−1^ is the stretching vibration peak of C-H, and the peaks of C=O, C=C and C-O are located at ~1700 cm^−1^, ~1600 cm^−1^ cm, and ~1100 cm^−1^ respectively [24,25,26]. In addition to the C-H single bond, GO, SB, and SB/GO all have four kinds of bonds: O-H, C=C, C=O, and C-O. This is because GO itself is only composed of C and O and contains some hydroxyl and carboxylic groups at its edge, while SB comes from corn straw (mainly composed of lignin, cellulose, and hemicellulose [27]), and the C-H bond is not completely decomposed in the process of high temperature pyrolysis, but a part of it remains. The presence of O-H, C=C, C=O, and C-O indicates that the three materials all contain hydroxyl and carboxyl groups and other oxygen-containing functional groups, which can partially dissociate in water, and then adsorb complex heavy metal ions and other pollutants in the water.

### 3.2. Optimizing Operation Parameters 

**Figure 3:**
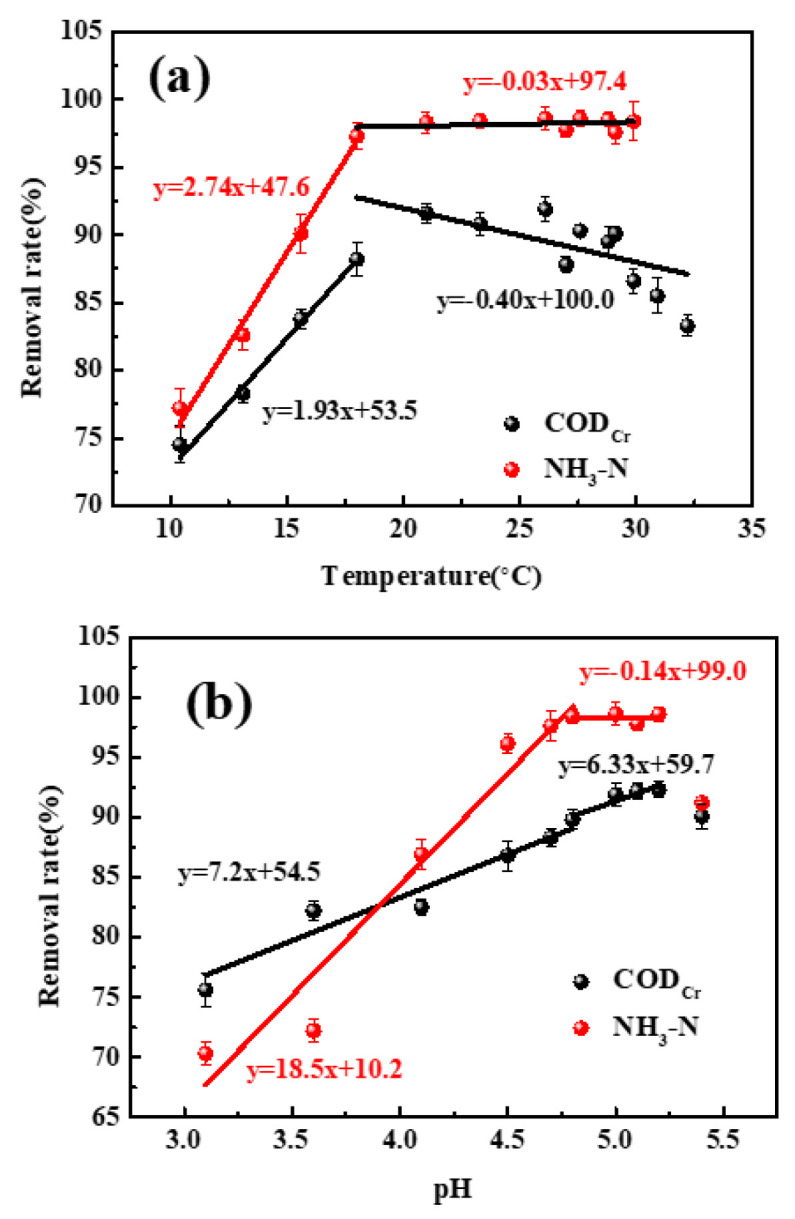
The effect of (**a**) temperature and (**b**) pH on removal of COD_Cr_ and NH_3_-N.

Previous studies showed that the pH and temperature of polluted water have a great effect on pollutant absorption using biochar [28]. The effects of pH and temperature on COD_Cr_ and NH_3_-N removal via the SGU system are shown in Figure 3a,b. As is known, the absorption process is endothermic. Therefore, it is found that the COD_Cr_ and NH_3_-N removal rate increases with the temperature increasing from 10 °C to 21 °C (Figure 3a). When 21 °C < temperature < 30 °C, the removal rate of NH_3_-N (>95%) and COD_Cr_ (>87%) remains unchanged. Furthermore, as the temperature increases continually, the removal rate of COD_Cr_ decreases, which can be attributed to molecular thermal motion acceleration and the pollutants’ desorption from the surface of SB/GO with the temperature increasing.

The effect of the pH is shown Figure 3b. On the whole, the change trend of the removal rate of NH_3_-N is similar to that of COD_Cr_. When the pH changed from 3.1 to 4.8, the removal rate increased continually, and then it remained stable (4.8 < pH < 5.2). As mentioned above, negatively charged functional groups grow on the biochar surface. Therefore, H^+^ can be absorbed on the surface of SB/GO, i.e., some active sites on the surface of SB-GO can be consumed by H^+^, which hinders the absorption of pollution [29]. An increase in pH can make active sites activate again and increase the absorption rate. For a pH > 5.2, the removal rate decreased. The phenomenon may be caused by the effect of pH on the structure of pollutants. Based on these facts, the temperature and pH of polluted water were adjusted to 21~30 °C and 4.8~5.2.

### 3.3. Removal Performance Comparation

#### 3.3.1. COD_Cr_ Removal

**Figure 4:**
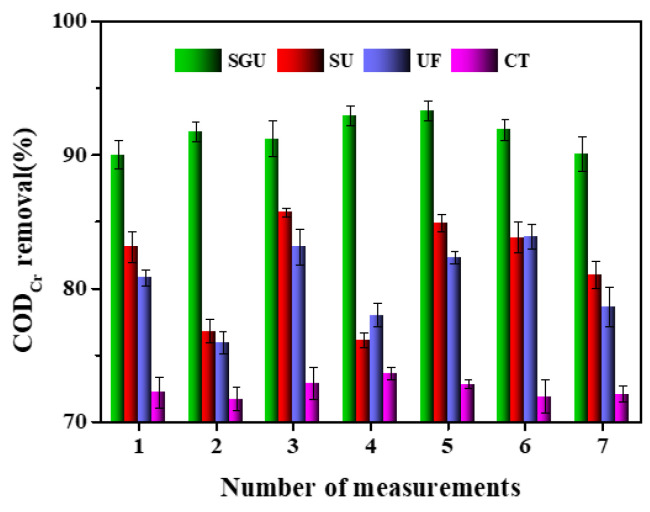
Comparation of COD_Cr_ removal performance of different methods.

According to Figure 4, under the optimal temperature and pH conditions, the COD_Cr_ removal rate of SGU is 89.8–92.8%, and the average removal rate is 91%. Compared with SU, UF and CT, the removal performance of the SGU process is better. The COD_Cr_ removal efficiency of UF is lower than that of SGU and SU but higher than that of CT. It is shown that some pollutants of a big size can be removed by UF, but small and dissolved matter cannot be treated. Furthermore, the removal performance of SB-UF was enhanced slightly compared to UF, and the removal performance of SGU increased evidently. Although the UF membrane has a good retention rate of macromolecule hydrophilic substances, macromolecules can significantly reduce membrane flux. Therefore, when UF is used to purify wastewater, a pretreatment process should be added to remove most organic matter in the water. Because SB/GO is a high-performance composite material composed of two porous and fiber-rich substances, SB/GO is more suitable for UF membrane pretreatment. In addition, there are several micro-pores in SB/GO particles, which is equivalent to the transition holes of granular carbon. Water flowing through the pores increases the adsorption capacity of SB/GO, thus playing a mechanical interception role on macromolecular organic compounds. Meanwhile, the interception layer and concentration polarization layer are formed on the surface of SB/GO and UF. They have a certain thickness, which can play a role in multi-layer interception [17,18,19,20]. As can be seen from Table 2, there are significant differences in the removal of COD_Cr_ among the different methods in general.

In the test lasting several months, the effluent COD_Cr_ is stable at about 17 mg/L and better than the quality standard of domestic miscellaneous water of the Chinese Ministry of Construction (CJ25.1-89).

#### 3.3.2. NH_3_-N Removal

**Figure 5:**
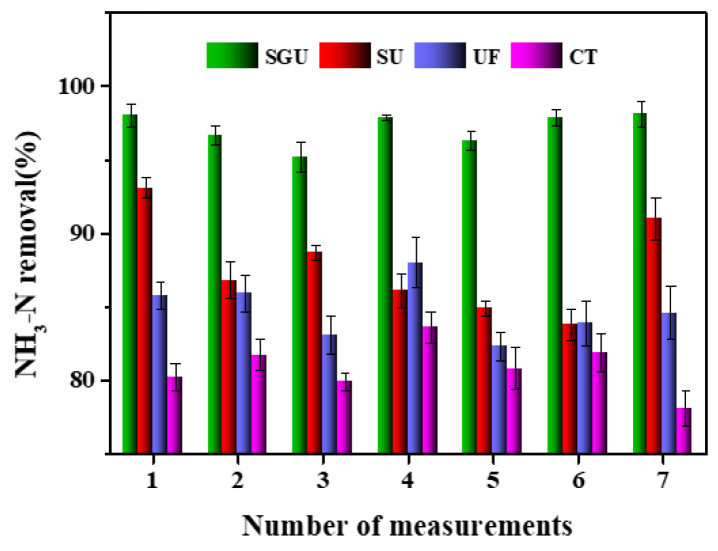
Comparation of NH_3_-N removal performance of different methods.

A comparation of the NH_3_-N removal performance of different methods is shown in Figure 5. In the range of the best basic conditions, SB-GO-UF has a good removal performance regarding NH_3_-N. The effluent NH_3_-N is 0.4~1.1 mg/L, and the removal rate is 96–99% (average 97%), which is about 10–13%, 6–8%, and 5–7% higher than that of CT, UF, and SU, respectively. It can also be seen that there is little difference between the UF and SU processes alone, indicating that the pretreatment effect of straw biological carbon on NH_3_-N is weak, and the pretreatment effect is significantly increased with the addition of GO. This may be caused by the specific three-dimensional interleaved network structure between the straw biological carbon and GO. As seen in Table 3, there are significant differences in the removal of NH_3_-N among the different methods in general. Within the range of optimal conditions, the ammonia nitrogen concentration of effluent water of the system is better than the quality standard of domestic miscellaneous water of the Ministry of Construction of China (CJ25.1-89).

#### 3.3.3. Turbidity Removal

**Figure 6:**
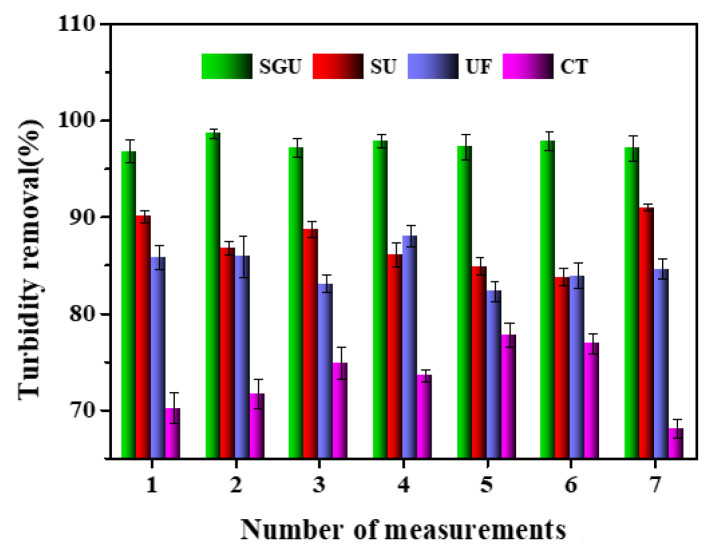
Comparation of turbidity removal performance of different methods.

A comparation of the turbidity removal performance of different methods is shown in Figure 6. The influent turbidity of the system is relatively high and fluctuates greatly. At the beginning of the operation, since the gel layer on the membrane surface had not yet formed, the membrane had a poor retention effect on small molecular colloidal substances, resulting in the turbidity of the membrane effluent changing greatly with the change in influent turbidity and the extension of the operation time [6,13]. The gel layer is formed on the membrane surface, which makes the actual filtration aperture of the membrane smaller and enhances the membrane’s retention of tiny colloidal substances, thus keeping the turbidity of the membrane effluent below 1 NTU, which is better than the Chinese Ministry of Construction water quality standard (CJ25.1-89). In addition to the excellent turbidity removal effect of SGU, the turbidity removal performance of UF and SU is ideal, which is obviously higher than that of the conventional process. There is no significant difference in the turbidity removal rate between UF and SU, indicating that turbidity removal occurs mainly through membrane interception, and the addition of SB cannot significantly improve the turbidity removal rate of UF. Additionally, SGU itself has a very large specific surface area, and the adsorption of turbidity substances is very sufficient (98%). The turbidity removal performance is reasonable. Except for traditional processes, all the other methods can achieve effluent turbidity standards. Meantime, as seen in Table 4, there are significant differences in the removal of turbidity among the different methods in general.

#### 3.3.4. Pb^2+^ Removal

**Figure 7:**
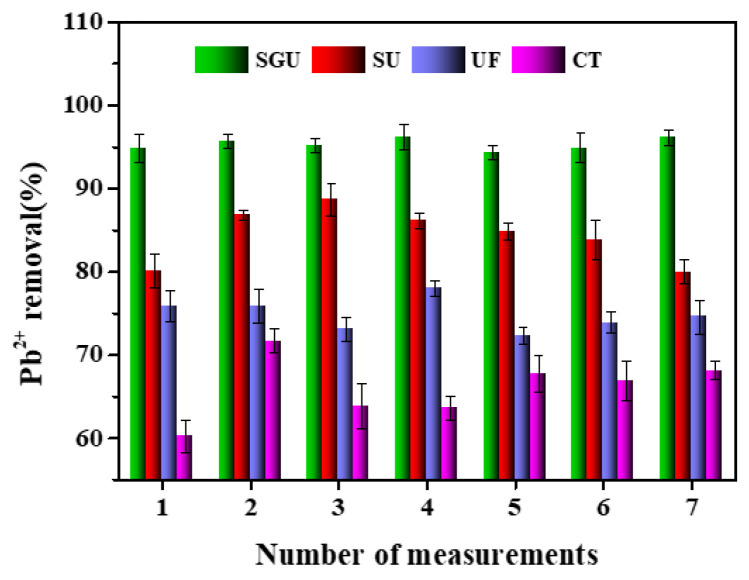
Comparation of Pb^2+^ removal performance of different methods.

A comparation of the Pb^2+^ removal performance of different methods is shown in Figure 7. In the experiment, it was found that SB and GO themselves had a significant effect on the removal of Pb^2+^. In the range of the optimal basic conditions (temperature and pH), SGU has a good removal effect on Pb^2+^. The effluent Pb^2+^ is 0.05–0.4 mg/L, and the removal rate of Pb^2+^ is 94–97% (average 95%), which is 18–33%, 15–28%, and 6–12% higher than CT, UF, and SU. It can also be seen that there is little difference between UF and CT, indicating that UF has a mediocre pretreatment effect on Pb^2+^, and the pretreatment effect is significantly increased after the introduction of GO. Because the surface of biochar contains a variety of negatively charged surface functional groups, these functional groups can form specific complexes with heavy metal ions in wastewater through coordination, which can anchor heavy metal ions on the surface of biochar. At the same time, the negatively charged oxygen-containing functional groups introduced into the edges and surface defects of GO are just combined with the negatively charged biochar complement, which can be used as the adsorption site to form a complex with heavy metal ions. The removal efficiency of heavy metal ions is greatly improved [13,14,15,16]. As seen in Table 5, there are significant differences in the removal of Pb^2+^ among the different methods in general. Within the range of optimal conditions, the Pb^2+^ concentration of effluent water of the system is better than the quality standard of domestic miscellaneous water of the Ministry of Construction of China (CJ25.1-89).

The T-tests for pollutant removal by different methods are shown in Table 6. It can be seen that although different treatment methods for all pollutants have significant differences in general, there is no significant difference between the UF and SU methods for COD_Cr_ removal, and the reasons for this have been discussed above. For the other pollution removal methods, the overall and local differences, that is, the overall and local consistencies, are significant.

### 3.4. Membrane Contamination and Cleaning

**Figure 8:**
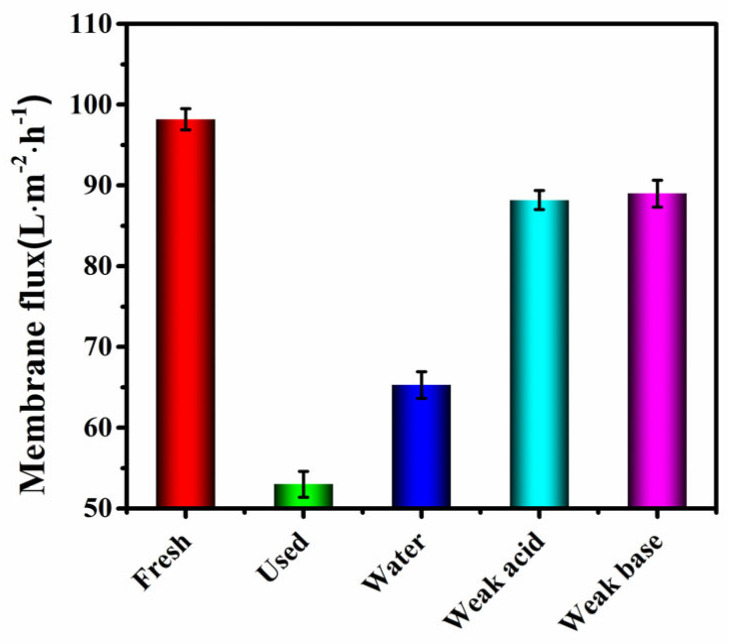
Comparation of membrane flux washed with different conditions.

When the SGU system is running, the osmotic pressure of the membrane is positively correlated with time, and the membrane flux is negatively correlated with time, which causes membrane pollution. Therefore, the influence of different backwashing conditions (water backwashing, weak acid backwashing, and weak alkali backwashing) on membrane flux was studied. Clean water washing entails direct backwashing with clean water; weak pickling refers to soaking in 0.8–1.2% HC1 or citric acid solution for 25 min and then backwashing with water; and weak alkali washing means that after backwashing with water, soak in 0.8–1.2% NaOH solution for 25 min, and then backwash with water. As shown in Figure 8, the recovery rates of the membrane flux under clear water, weak acid and weak base backwashing conditions were 65%, 88%, and 89%, respectively. The use of weak pickling film has the best effect, but the damage to the film is greater, so this system uses weak alkali washing. Regular cleaning of the membrane can effectively remove membrane pollution, increase flux, reduce energy consumption, and thus improve the efficiency of sewage treatment [30].

## 4. Conclusions

In order to solve the problem of low treatment efficiency of wastewater containing heavy metals in mining areas, SGU was formed by combining SB, GO, and UF to treat polluted water in a mining area. The pH and temperature of polluted water parameters of the process during operation were optimized. The removal effects of SGU, SU, UF, and CT were compared, and the effects of clean water, weak acid, and weak alkali backwashing methods on membrane regeneration were investigated, and the following conclusions were reached:

(1) When the pH and temperature of polluted water were set at 4.8–5.2 and 21–30 °C, respectively, the SGU process had the best removal effect on COD_Cr_ and NH_3_-N.
(2) The introduction of SB and GO has a significant effect on the improvement in effluent quality. The average removal rates of COD_Cr_, NH_3_-N, turbidity, and Pb^2+^ by the combined GO-MBR process were 91%, 97%, 98%, and 95%, respectively, which were higher than SU, UF, and CT. The water quality is better than the quality standard of the domestic miscellaneous water of the Ministry of Construction of China (CJ25.1-89).
(3) The weak alkali backwashing method can restore the membrane flux to 89% of the new membrane, so the system should use weak alkali washing.

This study is expected to provide scientific and theoretical support for the advanced treatment of polluted water with more unique substances in mining areas.

## Figures and Tables

**Table 1:** The parameters of the raw water.

Parameters	Values	Methods/Equipments
COD_Cr_ (mg·L^−1^)	326~572	Dichromate titration
NH_3_-N (mg·L^−1^)	34.9~79	Nasser’s reagent spectrophotometry
Turbidity (NTU)	48~177	Turbidity meter (WZB-182)
Pb^2+^ (mg·L^−1^)	23–31	Spectrophotometry
Temperature (°C)	10~31	Thermometer
pH	6~9	pH meter (PB-10)
DO (mg/L)	1~6	Dissolved oxygen analyzer (Polymetron 9582)

**Table 2:** ANOVA of COD_Cr_ removal via different methods.

	Sum of Square	DF	Mean Square	*F*-Value	*p*-Value
Model	3880.5	3	1293.5	151.7	0
Error	682.2	80	8.5		
Total	4562.7	83			

Notes: *p* < 0.05 indicates that the treatments reached a significant level.

**Table 3:** ANOVA of NH_3_-N removal via different methods.

	Sum of Square	DF	Mean Square	*F*-Value	*p*-Value
Model	3029.8	3	1009.9	126.6	0
Error	638.1	80	8.0		
Total	3667.9	83			

Notes: *p* < 0.05 indicates that the treatments reached a significant level.

**Table 4:** ANOVA of turbidity removal via different methods.

	Sum of Square	DF	Mean Square	*F*-Value	*p*-Value
Model	6247.4	3	2082.5	220.8	0
Error	754.3	80	9.4		
Total	7001.7	83			

Notes: *p* < 0.05 indicates that the treatments reached a significant level.

**Table 5:** ANOVA of Pb^2+^ removal via different methods.

	Sum of Square	DF	Mean Square	*F*-Value	*p*-Value
Model	9986.9	3	3329.0	222.7	0
Error	1195.9	80	14.9		
Total	11,182.8	83			

Notes: *p* < 0.05 indicates that the treatments reached a significant level.

**Table 6:** T-test of different pollutant removal via different methods.

Pollutants	SU-SUG (Sig.)	UF-SUG (Sig.)	UF-SU (Sig.)	CT-SUG (Sig.)	CT-SU (Sig.)	CT-UF (Sig.)
CODcr	1	1	0	1	1	1
NH_3_-N	1	1	1	1	1	1
Turbidity	1	1	1	1	1	1
Pb^2+^	1	1	1	1	1	1

Notes: Sig. = 1 indicates that the difference in the means is significant at the 0.05 level; Sig. = 0 indicates that the difference in the means is not significant at the 0.05 level.

## Data Availability

The datasets used and/or analyzed during the current study are available from the corresponding author on reasonable request.
